# Variation in Root-Associated Microbial Communities among Three Different Plant Species in Natural Desert Ecosystem

**DOI:** 10.3390/plants13172468

**Published:** 2024-09-03

**Authors:** Yulin Zhang, Yi Du, Zhihao Zhang, Waqar Islam, Fanjiang Zeng

**Affiliations:** 1 College of Ecology and Environmental, Xinjiang University, Urumqi 830046, China; zhangyl20201051213@163.com; 2 Xinjiang Key Laboratory of Desert Plant Roots Ecology and Vegetation Restoration, Xinjiang Institute of Ecology and Geography, Chinese Academy of Sciences, Urumqi 830011, China; duyi1996002001@163.com (Y.D.); zhangzh@ms.xjb.ac.cn (Z.Z.); 3 State Key Laboratory of Desert and Oasis Ecology, Key Laboratory of Ecological Safety and Sustainable Development in Arid Lands, Xinjiang Institute of Ecology and Geography, Chinese Academy of Sciences, Urumqi 830011, China; 4 Cele National Station of Observation and Research for Desert-Grassland Ecosystems, Cele 848300, China; 5 University of Chinese Academy of Sciences, Beijing 100049, China

**Keywords:** desert plants, microbial communities, soil nutrients, arid environments, rhizosphere soil

## Abstract

The process and function that underlie the assembly of root-associated microbiomes may be strongly linked to the survival strategy of plants. However, the assembly and functional changes of root-associated microbial communities in different desert plants in natural desert ecosystems are still unclear. Thus, we studied the microbial communities and diversity of root endosphere (RE), rhizosphere soil (RS), and bulk soil (BS) among three representative desert plants (*Alhagi sparsifolia*, *Tamarix ramosissima*, and *Calligonum caput-medusae*) in three Xinjiang desert regions {Taklimakan (CL), Gurbantünggüt (MSW), and Kumtag (TLF)} in China. This study found that the soil properties {electrical conductivity (EC), soil organic carbon (SOC), total nitrogen (TN) and phosphorus (TP), available nitrogen (AN) and phosphorus (AP)} of *C. caput-medusae* were significantly lower than those of *A. sparsifolia* and *T. ramosissima*, while the root nutrients (TN and TP) of *A. sparsifolia* were significantly higher compared to *C. caput-medusae* and *T. ramosissima*. The beta diversity of bacteria and fungi (RE) among the three desert plants was significantly different. The common OTU numbers of bacteria and fungi in three compartments (RE, RS, and BS) of the three desert plants were ranked as RS > BS > RE. The bacterial and fungal (RE) Shannon and Simpson indexes of *C. caput-medusae* were significantly lower as compared to those of *A. sparsifolia* and *T. ramosissima*. Additionally, bacterial and fungal (RE and RS) node numbers and average degree of *C. caput-medusae* were lower than those found in *A. sparsifolia* and *T. ramosissima*. Root and soil nutrients collectively contributed to the composition of root-associated bacterial (RE, 12.4%; RS, 10.6%; BS, 16.6%) and fungal communities (RE, 34.3%; RS, 1.5%; BS, 17.7%). These findings demonstrate variations in the bacterial and fungal populations across different plant species with distinct compartments (RE, RS, and BS) in arid environments. More importantly, the study highlights how much soil and plant nutrients contribute to root-associated microbial communities.

## 1. Introduction

A close reciprocal interplay exists between the microbial assemblage and the plant host, serving as a crucial factor in maintaining well-being and enhancing the yield of the plant [1,2]. Nevertheless, the root serves as the primary organ for nutrient acquisition in plants, facilitating direct interactions with soil microbes and the formation of symbiotic relationships with bacteria and fungi [3,4]. The formation of the rhizosphere is primarily facilitated through the root-induced modification of the soil’s physical architecture [4]. Plants release substances from their roots into the surrounding soil, which help to draw in and support microbial communities [5,6,7]. Diverse compositions of rhizo-deposits enable plants to shape rhizosphere microbial communities for their benefit [8,9]. Consequently, the rhizosphere functions as a focal point for microbial activity, nutrient circulation, and the transformation of organic substances [4,5,7,9].

The assemblage and heterogeneity of microbial communities within arid soils are affected by the presence of distinct plant species and seasonal environmental changes [10]. The fluctuation of soil microbial functional groups throughout the seasons is intricately intertwined with plant diversity, climatic factors, soil nutrient levels, and various other relevant elements [11]. Soil properties (total nitrogen (TN), available phosphorus (AP), and pH) play a significant role in determining how plant and soil microbial communities adapt to alterations in their surroundings [12,13,14]. The composition of soil bacterial communities was mainly impacted by the soil total phosphorus (TP), and a discernible trend of diminishing bacterial diversity was observed with increasing distance, suggesting that environmental factors exert a more substantial impact on the shifts in bacterial communities than geographical proximity within the Gurbantünggüt desert [15]. An investigation examining the bacterial taxa in the arid regions of central Mexico revealed that the relative abundance of some taxa (e.g., *Actinomycetota*, *Pseudomonadota*, and *Acidobacteriota*) accounted for a large proportion in the rhizosphere of cactus species [16,17].

In extreme arid desert ecosystems, the spatial distribution of desert plants is affected by the availability of soil nutrients and water [18]. However, compared to the interspace area, desert vegetation had a faster nutrient turnover [19]. These canopy patches can extremely easily form a cool microclimate [20,21]. Because they have higher and faster nutrient cycling and greater microbial turnover, they are described as ‘fertile islands’, which may serve to form the structural and functional dynamics of desert environments and slow down the desertification process [22,23]. Different plant species often form ‘dunes’ of varying magnitude, and these dunes usually have different degrees of fertile island effect in desert ecosystems [24]. Therefore, species-specific vegetation (e.g., *Tamarix chinensis* and *Salix psammophila*) could be used to reasonably explain variations in ‘fertile island’ formation and discrepancies between soil properties beneath the canopy versus the interspace area [23,25]. Research has indicated that the ‘fertile islands’ within the Taklimakan desert are consistent with canopy size, with *T. ramosissima* exhibiting the highest level of soil fertility, followed by *Karelinia caspia* and *A. sparsifolia* [26]. Also, it is suggested that the influence of fertile islands on microbial energy strategies and life-history strategies via soil organic carbon (SOC) availability could be one of the mechanisms that shape the spatial heterogeneity of soil and root microbial communities in desert plants [27]. Additionally, altered energy and life-history strategies may further affect the decomposition activity of microorganisms in soil labile and stable carbon pools [27,28].

Desert ecosystems are particularly harsh environments, characterized by low moisture availability, high temperatures, and limited nutrient resources [29,30]. Microbes from extreme environments, including bacteria and fungi, display unique genetic and physiological characteristics that allow them to survive in challenging conditions [31,32,33]. The Taklimakan, Gurbantünggüt, and Kumtag deserts are acknowledged for their natural formation as severe environmental conditions, marked by aridity, salinization, and elevated temperatures, which serve as the primary non-living stress factors [30,34,35]. Despite these challenges, certain plant species have adapted to thrive in these harsh environments, in part through symbiotic associations with specific microbial communities in their root systems [36,37,38]. The composition and diversity of the root-associated microbial communities are deemed essential for facilitating plant–microbial interactions, thereby ultimately shaping the growth and development of vegetation in arid environments [36,39,40]. *A. sparsifolia*, *T. ramosissima*, and *Calligonum caput-medusae* are key desert flora that contribute significantly to maintaining balance in desert environments, promoting biodiversity, impacting climate control, and containing healing properties [26,41,42]. Our goal was to understand how different plant species affect the diversity of root-associated microbial communities and their potential function in dry environments. Our hypothesis posits two key assertions: Firstly, we propose that the roots of various desert plants host a core microbiota, consisting of both bacterial and fungal assemblages, which collaboratively facilitate adaptation to the harsh arid conditions prevalent in desert environments. Secondly, we anticipate that the factors influencing the composition and diversity of these microbial communities (RE, RS, and BS) differ significantly across distinct desert plant species, thereby shaping unique bacterial and fungal community structures within their root systems.

## 2. Results

### 2.1. Variations in Soil and Root Nutrients among Three Desert Plants

*C. caput-medusae* exhibited significantly lower levels of soil physical and chemical properties (SOC, TN, TP, AN, AP, and EC) in comparison to *A. sparsifolia* and *T. ramosissima* (one-way ANOVA; Table 1). In contrast, soil TK content did not show significant variation among the three desert plants (one-way ANOVA; Table 1). Root nutrients (ROC, TN, TP, and TK) of *T. ramosissima* were found to be significantly lower than those of *A. sparsifolia* and *C. caput-medusae* (one-way ANOVA; Table 2). The height and crown width of *A. sparsifolia* were significantly lower compared to *T. ramosissima* and *C. caput-medusae* (Table S1). The soil nutrients (TP, TK, AN, AP, and AK) in three desert plants were higher than those in bare soil (Table 1 and Table S2).

### 2.2. Sequencing and OTU Number of Root-Associated Microbes among Three Desert Plants

**Figure 1:**
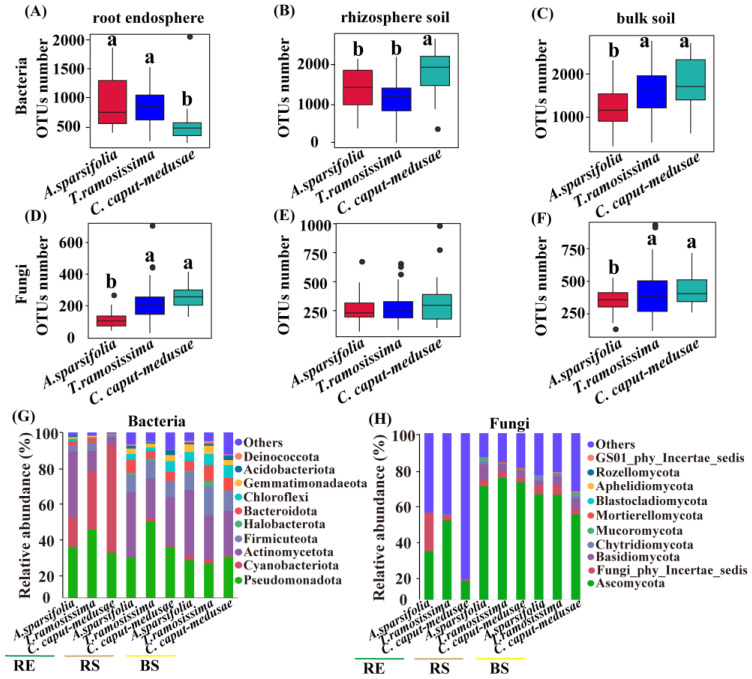
The OTU number (bacteria and fungi) and relative abundance {dominant bacteria and fungi taxa (top 10 phyla)} of root endosphere (RE), rhizosphere soil (RS), and bulk soil (BS) in three desert plants. Different lowercase letters (a and b) indicate significant differences among species at the *p* < 0.05 level (ANOVA and Duncan’s test). (**A**–**C**) OTUs number of the bacteria, (**D**–**F**) OTUs number of the fungi, and (**G**) relative abundance of dominant bacteria and (**H**) relative abundance of dominant fungi.

Plant species and different compartments had significant effects on the sequencing and OTU number (bacteria and fungi) (two-way ANOVA; Table 3). Furthermore, the interaction of various species and compartments significantly impacted the sequencing (bacteria and fungi) and OTU number (bacteria), while no significant effect was observed on the OTU number (fungi) (two-way ANOVA; Table 3). *C. caput-medusae* had a significantly lower OTU number of bacterial communities (RE) compared to *A. sparsifolia* and *T. ramosissima*, but the bacterial communities (RS) of *C. caput-medusae* had a significantly higher OTU number compared to *A. sparsifolia* and *T. ramosissima* (Figure 1A,B). Furthermore, *A. sparsifolia* exhibited a significantly lower OTU number of bacterial communities (BS) compared to *C. caput-medusae* and *T. ramosissima* (Figure 1C). Additionally, the fungal communities (RE and BS) of *A. sparsifolia* had a significantly lower OTU number compared to *C. caput-medusae* and *T. ramosissima* (Figure 1D,F). There was no significant difference in the OTU number among three desert plants (Figure 1E). The relative abundance of *Pseudomonadota* (bacterial taxa) in the RE and RS of *T. ramosissima* was found to be higher compared to *A. sparsifolia* and *C. caput-medusae* (Figure 1G). Moreover, the relative abundance of *Ascomycota* (fungal taxa) in RS and BS was higher compared to that in the RE among all three desert plants (Figure 1H). In addition, the sequencing number (bacteria) of RE was higher than that of RS and BS among three desert plants, whereas the sequencing number (fungi) of RE was lower than that of RS and BS in two desert plants (*T. ramosissima* and *C. caput-medusae*) (Figure S1A,B).

### 2.3. Alpha Diversity of Root-Associated Microbes among Three Desert Plants

**Figure 2:**
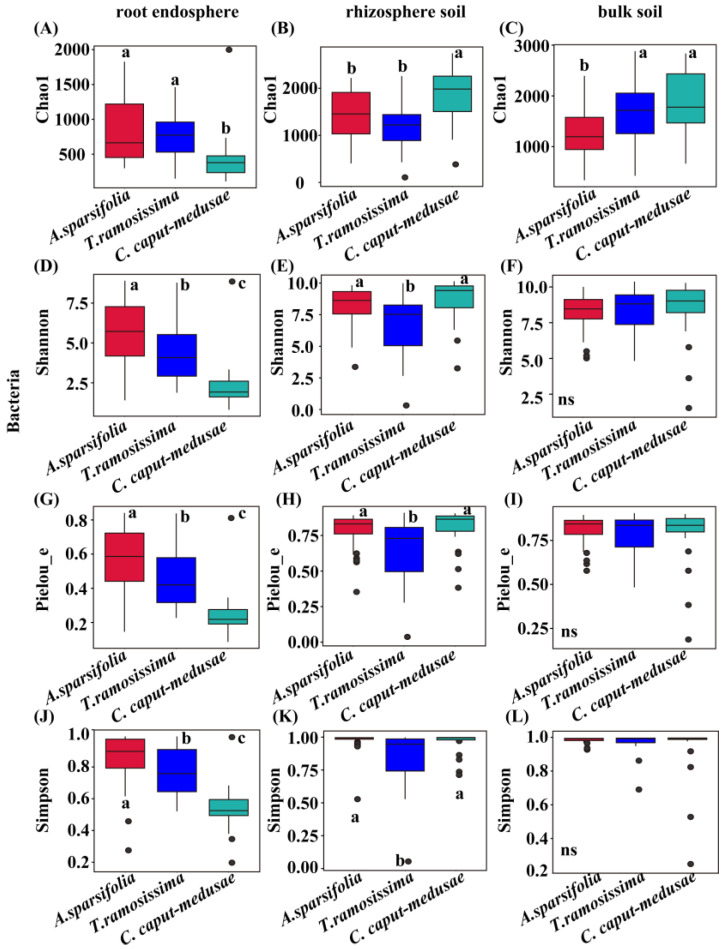
Alpha diversity {(**A**–**C**) Chao1, (**D**–**F**) Shannon, (**G**–**I**) Pielou_e, and (**J**–**L**) Simpson indexes} of root endosphere (RE), rhizosphere soil (RS), and bulk soil (BS) bacteria in three desert plants. Different lowercase letters (a–c) indicate significant differences among species at the *p* < 0.05 level and the ns indicate no significant differences among species at the *p* > 0.05 level (ANOVA and Duncan’s test).

Bacteria (RE) in *C. caput-medusae* exhibited significantly lower alpha diversity (Chao1, Shannon, Pielou_e, and Simpson index) compared to *A. sparsifolia* and *T. ramosissima* (Figure 2A,D,G,J). However, the bacterial (RS) Chao1, Shannon, Pielou_e, and Simpson indexes of *C. caput-medusae* were significantly higher than those of *T. ramosissima* (Figure 2B,E,H,K). The bacterial (BS) Chao1 index in *A. sparsifolia* was significantly lower than that of *C. caput-medusae* and *T. ramosissima* (Figure 2C). Moreover, other bacterial (BS) Shannon, Pielou_e, and Simpson indexes in the three desert plants were not significantly different (Figure 2F,I,L).

The fungal (RE) Chao1 index in *A. sparsifolia* was significantly lower than that of *C. caput-medusae* and *T. ramosissima* (Figure S2A). Other fungal (RE) alpha diversity (Shannon, Simpson, and Pielou_e indexes) in *T. ramosissima* was significantly higher than that of *C. caput-medusae* (Figure S2D,G,J). The fungal (RS and BS) Chao1, Shannon, Pielou_e, and Simpson indexes in the three desert plants were not significantly different (Figure S2B,C,E,F,H,I,K,L).

### 2.4. Beta Diversity of Root-Associated Microorganisms among Three Desert Plants

**Figure 3:**
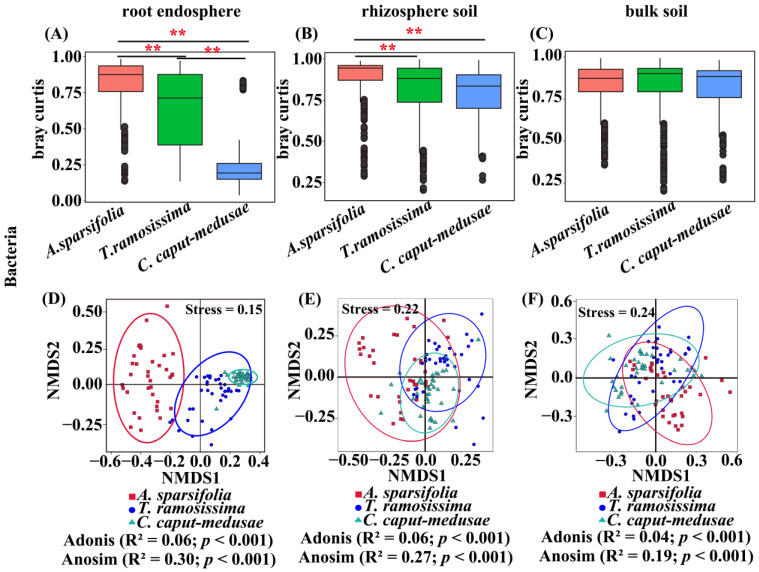
Beta diversity {(**A**–**C**) Bray–Curtis and (**D**–**F**) nonmetric multidimensional scaling} of root endosphere (RE), rhizosphere soil (RS), and bulk soil (BS) bacteria of three desert plants. ** *p* < 0.01.

The bacterial (RE) beta diversity among the three desert plants was significantly different (Figure 3A). However, there was no significant difference in the bacterial (BS) beta diversity among three desert plants (Figure 3C). The bacterial (RS) beta diversity in *A. sparsifolia* was significantly different than that of *T. ramosissima* and *C. caput-medusae*, but the bacterial (RS) beta diversity in *T. ramosissima* was not significantly different than *C. caput-medusae* (Figure 3B). The fungal (RE) beta diversity of the three desert plants was significantly different (Figure S3A). The fungal (RS and BS) beta diversity in *T. ramosissima* was significantly different than that in *C. caput-medusae* (Figure S3B,C).

### 2.5. Core and Differential Microbiota of Root-Associated Microbes among Three Desert Plants

**Figure 4:**
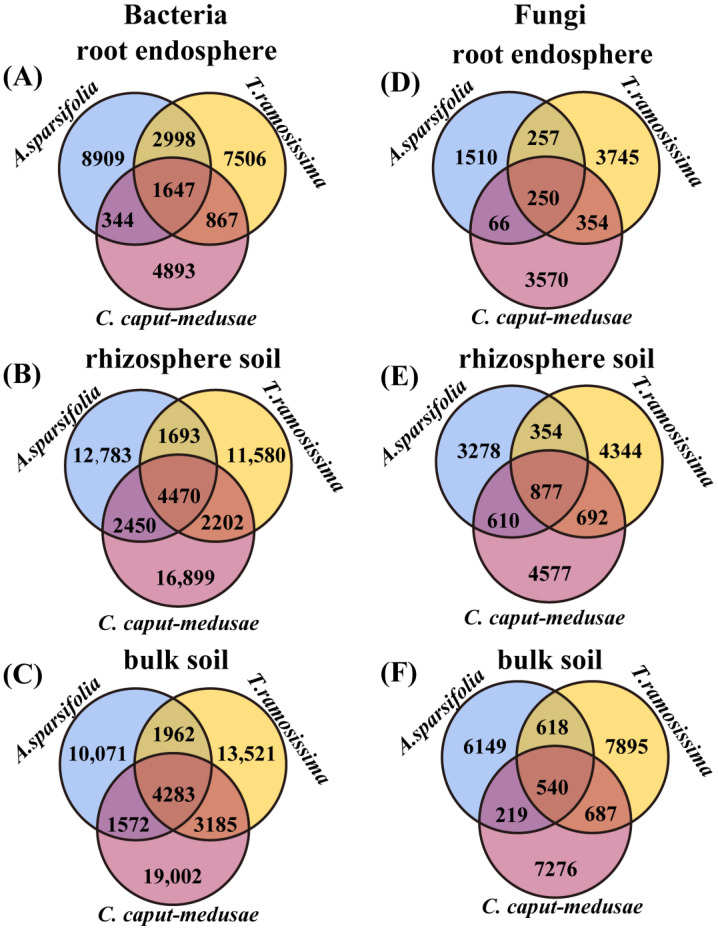
Core and differential microbiota {(**A**–**C**) OTUs number of the bacteria and (**D**–**F**) OTUs number of the fungi} of root endosphere (RE), rhizosphere soil (RS), and bulk soil (BS) bacteria and fungi among three desert plants.

The bacterial and fungal communities (RE, RS, and BS) of *A. sparsifolia* had 8909 and 1510, 12,783 and 3728, and 10,071 and 6149 unique OTUs, respectively (Figure 4A–F). The bacterial and fungal communities (RE, RS, and BS) of *T. ramosissima* had 7506 and 3745, 11,580 and 4344, and 13,521 and 7895 unique OTUs, respectively (Figure 4A–F). The bacterial and fungal communities (RE, RS, and BS) of *C. caput-medusae* had 4893 and 3570, 16,899 and 4577, and 19,002 and 7276 unique OTUs, respectively (Figure 4A–F). A total of 1647 and 250, 4470 and 877, and 4283 and 540 core OTUs were stable in the bacterial and fungal communities (RE, RS, and BS) among the three plants, respectively (Figure 4A–F).

### 2.6. LEfSe Analysis of Root-Associated Microbes among Three Desert Plantss

**Figure 5:**
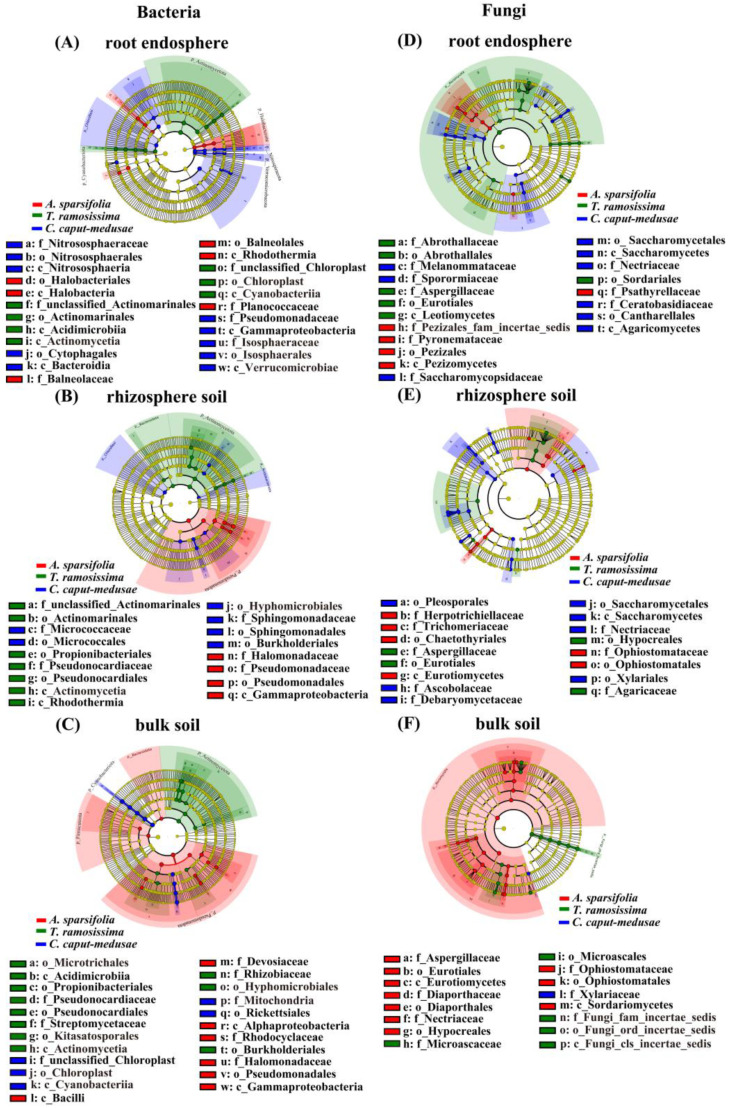
Linear discriminant analysis effect size (LEfSe) {(**A**–**C**) LEfSe analysis of the bacteria and (**D**–**F**) LEfSe analysis of the fungi} of root endosphere (RE), rhizosphere soil (RS), and bulk soil (BS) bacteria and fungi among three desert plants.

Through LEfse analysis, the taxonomic groups *Halobacterota* and *Pseudomonadota* were determined to be indicative of RE and RS (bacterial communities) linked to *A. sparsifolia* (Figure 5A,B). Additionally, *Firmicuteota*, *Bacteroidota*, and *Pseudomonadota* were identified as the biomarkers of BS (bacterial communities) in *A. sparsifolia* (Figure 5C). Conversely, the taxonomic groups *Actinomycetota* and *Cyanobacteriota* were recognized as biomarkers for RE (bacterial communities) in *T. ramosissima* (Figure 5A). Additionally, *T. ramosissima* showed *Bacteroidota* and *Actinomycetota* as biomarkers for RS (bacterial communities) (Figure 5B), with *Actinomycetota* acting as the indicator for BS (bacterial communities) (Figure 5C). *Nitrospiraeota* and *Verrucomicrobaeota* were recognized as biomarkers for RE (bacterial communities) in *C. caput-medusae* (Figure 5A), whereas *Chloroflexi* and *Cyanobacteriota* were determined to be biomarkers for RS and BS (bacterial communities) (Figure 5B,C). Moreover, *Ascomycota* and *Fungi_phy_Incertae_sedis* were identified as biomarkers for RE and BS (fungal communities) in *T. ramosissima*, while *A. sparsifolia* showed *Ascomycota* as a biomarker for BS (fungal communities) (Figure 5D–F).

### 2.7. Network of Root-Associated Microbes among Three Desert Plant Species

**Figure 6:**
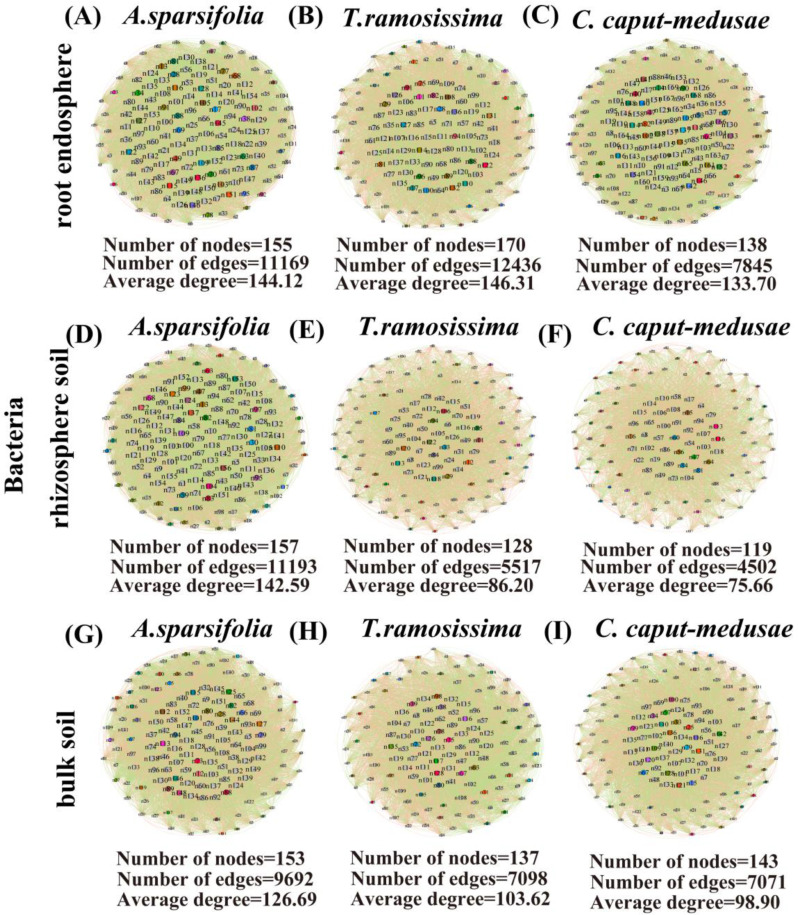
Co-occurrence network {(**A**,**D**,**G**) Network characteristics of the *A. sparsifolia*, (**B**,**E**,**H**) Network characteristics of the *T. ramosissima*, and (**C**,**F**,**I**) Network characteristics of the *C. caput-medusae*} of root endosphere (RE), rhizosphere soil (RS), and bulk soil (BS) bacteria of three desert plants.

The bacterial (RE and RS) network characteristics (nodes, edges, and average degree) of *C. caput-medusae* were discovered to be lower than those of *A. sparsifolia* and *T. ramosissima* (Figure 6A–F). However, the bacterial and fungal (BS) network characteristics (nodes, edges, and average degree) of *A. sparsifolia* were higher than *T. ramosissima* and *C. caput-medusae* (Figure 6G–I; Figure S5H,I). The fungal (RE) network characteristics (nodes, edges, and average degree) of *T. ramosissima* were lower than those of *A. sparsifolia* and *C. caput-medusae* (Figure S4A–C). In *C. caput-medusae*, the fungal (RS) network properties (nodes, edges, and average degree) were determined to be lower than those of *A. sparsifolia* and *T. ramosissima* (Figure S4D–F). Similarly, the fungal (BS) network characteristics (nodes, edges, and average degree) of *A. sparsifolia* were higher than those of *C. caput-medusae* and *T. ramosissima* (Figure S4G–I).

### 2.8. The Influence of Soil and Root Nutrients on the Root-Associated Microbial Communities among Three Desert Plants

**Figure 7:**
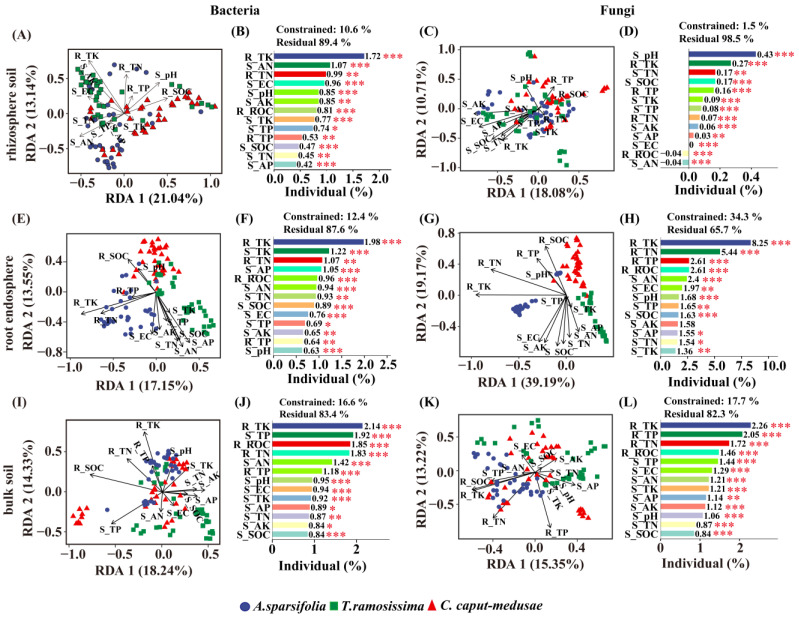
The main drivers of different bacterial and fungal communities {root endosphere (RE), rhizosphere soil (RS), and bulk soil (BS)} at the OTU level. The RDA plots show soil and root nutrients that significantly affect bacterial and fungal communities, according to the reduced model with 999 permutations. The results of HP analysis indicated the relative importance of environmental factors (soil and root) on bacterial and fungal communities. The column diagram shows the individual effect of each environmental factor (from hierarchical partitioning). SOC, soil organic carbon (g·kg^−1^); ROC, root organic carbon (g·kg^−1^); EC, electrical conductivity (mS·cm^−1^); TN, total nitrogen (g·kg^−1^); TP, total phosphorus (g·kg^−1^); TK, total potassium (g·kg^−1^); AN, available nitrogen (mg·kg^−1^); AP, available phosphorus (mg·kg^−1^); AK, available potassium (mg·kg^−1^). {(**A**) Redundancy analysis and (**B**) HP analysis of the root endosphere, (**E**) Redundancy analysis and (**F**) HP analysis of the rhizosphere soil, and (**I**) Redundancy analysis and (**J**) HP analysis of the bulk soil} of the bacteria and {(**C**) Redundancy analysis and (**D**) HP analysis of the root endosphere, (**G**) Redundancy analysis and (**H**) HP analysis of the rhizosphere soil, and (**K**) Redundancy analysis and (**L**) HP analysis of the bulk soil} of the fungi. Note: Significance codes, “*” *p* < 0.05; “**” *p* < 0.01; “***” *p* < 0.001.

The interaction between root nutrients and soil physical and chemical properties influenced the root-associated bacterial (RE 12.4%; RS 10.6%; BS 16.6%) and fungal (RE 34.3%; RS 1.5%; BS 17.7%) communities differently, with root factors having a higher impact on fungal communities (RE and BS) than soil factors (Figure 7). Root TK contents had the highest contribution to root-associated bacterial (RE: 1.98%; RS: 1.72%; BS: 2.14%) and fungal communities (RE: 8.25%; BS: 2.26%). Soil pH had the greatest impact on fungal (RS) communities (0.43%), but the least on bacterial (RE) communities (0.63%) (Figure 7D,F). In comparison to bacterial and fungal communities (RE and RS), soil SOC contents had the smallest contribution to bacterial and fungal communities (BS) (Figure 7J,L). The contribution of soil AP contents to bacterial and fungal (RS) communities was also determined to be the smallest (Figure 7A,B). Similarly, soil TK contents were found to contribute the least to fungal (RE) communities (Figure 7H).

## 3. Discussion

### 3.1. Dynamic Changes in Root and Soil Nutrients among Three Desert Plants

Previous research findings have shown that the formation of ‘fertile islands’ under the shade of perennial desert plants helps slow down desertification that is crucial for maintaining the structure and function of desert ecosystems [19,43]. The desert plants in the Taklimakan desert showed a connection between the ‘fertile islands’ phenomenon and the size of the canopy, with *T. ramosissima* having the greatest impact followed by *K. caspia* and *A. sparsifolia* [26]. Moreover, soil microhabitats beneath shrubs, in conjunction with shrub characteristics, may play a role in supporting macro-fauna activities and enhancing soil nutrients and microbial diversity in shifting sand environments [44]. In this study, the results showed that soil properties (SOC, TN, TP, AN, AP, and EC) were significantly lower in *C. caput-medusae* when compared to *A. sparsifolia* and *T. ramosissima*. On the other hand, root nutrients (ROC, TN, TP, and TK) were significantly higher in *A. sparsifolia* and *C. caput-medusae* in comparison to *T. ramosissima*. Additionally, the height and crown width of *A. sparsifolia* were significantly lower compared to *T. ramosissima* and *C. caput-medusae*. This is inconsistent with the results of this study. Previous studies focused on the work at one site, and the results of this study were explored at a regional scale, which may be related to the differences brought about by the regional-scale environment [26,45]. In contrast, desert plants with small canopies are adept at forming a cool microclimate characterized by reduced wind and interference, resulting in lower temperatures and evaporation compared to other species (larger canopies) and bare areas [20,21]. As a result, these plants (small canopies) show higher nutrients, quicker element cycling, and greater microbial activity, providing important insights into the structure and function of ecosystems within arid desert environments [22,23]. *C. caput-medusa* surrounding soil is poor compared to the *T. ramosissima* although the root nutrient content is higher. This may be related to root exudates. Research has found that amino acids significantly influence the activity of nitrogenase and phosphatases, which may enhance the organic matter decomposition and thus improve nutrient recycling [46]. Different densities of *Haloxylon ammodendron* (single-plant, two-plant, and three-plant) planting revealed that the release of ecgonine, raucaffricine, and neohesperidin helps in recruiting *Sphingomonadales* and increasing soil nutrient availability at the expense of biomass [47]. Future work is needed to study root exudates among three desert plants and analyze their relationships with plants, soil, and microorganisms.

### 3.2. Variations in Root Microbial Communities and Diversity among Three Desert Plants

Plants’ growth and development result in variations among microbial assemblages linked to roots, which in turn affects plant adaptive capacity, including the capacity to diminish both biological and environmental stress factors [48,49,50]. Undeniably, the influence of these microbial assemblages on adaptability varies substantially among various species, thus playing a crucial role in regulating the plant’s capacity to withstand harsh environmental circumstances [32,51]. Studies have revealed that variations among thirty angiosperm species, with specific microbial communities (RE and RS) potentially influence interspecies competition [50,51]. Furthermore, notable changes were observed in microbial (RE and RS) communities and the diversity of desert plants, with alterations in species composition, different compartments (RE, RS, and BS), and soil nutrients [32,52,53]. Our research revealed that plant species and compartments (RE, RS, and BS) had a significant impact on the sequencing and OTU number. In *C. caput-medusae*, the OTU number and alpha diversity (Shannon and Simpson index) of bacteria and fungi (RE) were significantly lower in comparison to the other two desert plants (*A. sparsifolia* and *T. ramosissima*). The OTU number and Chao1 index of bacteria (RS) in *C. caput-medusae* were significantly higher compared to the other two species (*A. sparsifolia* and *T. ramosissima*). However, the OTU number and Chao1 index of bacteria and fungi (BS) of *A. sparsifolia* were significantly lower compared to the other two species (*C. caput-medusae* and *T. ramosissima*). Interestingly, *A. sparsifolia* forms the largest ‘fertile island’ effect, with high nutrient concentration but low microbial diversity in the RS and BS. Moreover, the RE microbial diversity of *A. sparsifolia* was increased. In contrast, the RE microbial diversity of the other two species (*C. caput-medusae* and *T. ramosissima*) diminished, with elevated RS and BS microbial diversity, fostering a complementary effect among distinct compartments (RE, RS, and BS) that aids in resilience against external environmental fluctuations in desert plants. One study found that in the ‘fertile island’ with higher SOC content, most microorganisms obtained energy mainly through the metabolism of organic matter, and these organic energy preferences were mostly eutrophic microorganisms. In the ‘fertile island’ with low organic carbon content, the proportion of groups obtaining energy by oxidizing inorganic trace gases increased, and the users of these trace gases were mostly oligotrophic microorganisms, which also indicated that the heterogeneity of SOC content caused by the ‘fertile island’ effect may affect microbial energy and life-history strategies [28]. Further, it was found that the establishment of fertile islands in the desert ecosystem shaped soil bacterial communities, affecting soil properties and plant–soil feedback loops [27]. Therefore, more studies are needed to verify the relationship between root microbial communities of desert plants, soil fertile island effect, and root exudates in extreme arid desert areas.

### 3.3. Network Stability and Phyla and Taxa Change Characteristics of Root Microbial Communities among Three Desert Plants

Moreover, the results revealed that the fungal and bacterial (RS) differential OTU numbers of *C. caput-medusae* were higher compared to *A. sparsifolia* and *T. ramosissima*. In contrast, the network characteristics (nodes, edges, and average degree) of the RS (bacterial and fungal communities) in *C. caput-medusae* were found to be lower than those of *A. sparsifolia* and *T. ramosissima*. Conversely, the bacterial and fungal (BS) differential OTU number of *A. sparsifolia* was determined to be less than that of *T. ramosissima* and *C. caput-medusae*, whereas the network properties (nodes, edges, and average degree) showed the opposite trend. The distinction between these species is readily apparent, indicating a robust capacity for co-existence among different species [32,50]. Reducing the OTU number in the three compartments (RE, RS, and BS) leads to a rise in network intricacy, while boosting the OTU number leads to a reduction in network intricacy. These results are intriguing, yet the variability in environmental conditions within desert regions introduces uncertainties regarding the survival of desert flora. The intimate association and synergistic impact between the microbial diversity within the root zones of various species underscore the mutual evolution of plants and their microenvironment, facilitating nutrient uptake or alleviating host stress, countering external environmental disturbances to desert flora, and securing the continuation of the plant population [30,38,54].

To acclimatize to the unique environmental circumstances prevalent in desert ecosystems, it is imperative for desert flora to harbor specialized microbial communities that enable them to harness resources efficiently [32,55,56,57]. Bacterial taxa (e.g., *Actinomycetota*, *Pseudomonadota*, *Bacteroidota*, and *Firmicuteota*) were recently discovered in the roots of *Haloxylon* [32]. Recent investigations have revealed a predominance of *Actinomycetota*, *Pseudomonadota*, and *Firmicuteota* within the rhizospheric soil of both *Halostachys caspica* and *Salicornia alterniflora* [58]. Additional phyla, including *Nitrospirae* and *Synergistetes*, were identified in the root-associated microorganisms of desert plants [57,58,59]. As the severity of drought intensifies, alterations in the aggregation process of soil microorganisms (bacteria and fungi) have been found, typically marked by an increase in *Actinomycetota* and *Chloroflexi* populations, while *Pseudomonadota* populations tend to decrease [60,61]. Desert plants typically inhabit arid and high-temperature environments, offering a unique habitat for microbial life, though significant variations in microbial species composition exist among different plant species [32,57,58,62]. Indeed, roots not only recruit microorganisms nearby but also have stable microbial species-specific genetic determinants [56]. This study used LEfSe analysis to find the root bacteria (*Halobacterota*, *Pseudomonadota*, *Bacteroidota*, and *Firmicuteota*) and fungi taxa (*Ascomycota*) in *A. sparsifolia*, the root bacteria taxa (*Actinomycetota*, *Cyanobacteriota*, and *Bacteroidota*) and fungi taxa (*Ascomycota* and *Fungi_phy_Incertae_sedis*) in *T. ramosissima*, and the root bacteria taxa (*Nitrospiraeota*, *Verrucomicrobaeota*, *Chloroflexi*, and *Cyanobacteriota*) in *C. caput-medusae*. Studies have shown that *Halobacterota* is extremely halophilic and *Actinomycetota* can live in an environment with extreme pH value, salinity, and nutrient scarcity [63,64]. *Pseudomonadota* produces diaminopimelic acid, a special component of the cell wall that resists environmental pressure [65]. It was also found that *Bacteroidota* can help to convert biomass-derived sugars into propionic acid, *Ascomycota* can use soluble carbohydrates, and *Nitrospiraeota* can biomineralize in the cell [66,67,68]. *Chloroflexi* may complete hydrolytic or oxidative degradation of various types of recalcitrant organic matter, including aromatic compounds (e.g., benzoate), polyaromatic hydrocarbons (e.g., fluorene), polychlorobiphenyl (e.g., 4-chlorobiphenyl), and organochlorine compounds (e.g., chloroalkanes, chlorocyclohexane) [69]. The variation in root-associated bacteria and fungi taxa among diverse desert deep-rooted plant species remains evident, facilitating enhanced absorption, conversion, and retention of essential nutrients for plant growth [32,57,70,71,72].

### 3.4. Effects of Environmental Factors on Root Microbial Communities among Three Desert Plants

The temporal fluctuations in certain taxa in both bulk and rhizosphere soils are influenced by soil nutrients, highlighting their significance as a key regulatory factor [73,74]. The soil factors explained 24.28% of the variability observed in the rhizosphere bacterial community structure of *Ferula sinkiangensis* [75]. In this study, the results suggest the total contribution of root and soil factors to impact the root-associated bacterial (RS 10.6%; RE 12.4%; BS 16.6%) and fungal (RS 1.5%; RE 34.3%; BS 17.7%) communities. The results indicate that soil-related factors and the way the host plant grows could play a crucial role in this phenomenon [76]. Additionally, plant nutrient acquisition strategies and soil nutrient status serve a crucial function in regulating plant rhizosphere effects on soil processes driven by microorganisms [3,77]. This study provides further evidence that local-scale factors, including microclimate, soil composition, and disturbance, may be more influential than broad-scale environmental factors in elucidating the drivers of plant community interactions [78]. Given the distinctive geographical attributes of desert ecosystems, such as climate variability, soil nutrient availability, and plant diversity, it is imperative for perennial deep-rooted desert plants to not only thrive autonomously for enhanced environmental resilience but also engage in mutualistic relationships with other species and symbiotic microorganisms to withstand unfavorable environmental conditions [36,41,79,80]. These results offer valuable illumination into the understanding of plants living in the desert, but follow-up research and monitoring are still needed.

## 4. Materials and Methods

### 4.1. Study Site Description and Sampling Design

**Figure 8:**
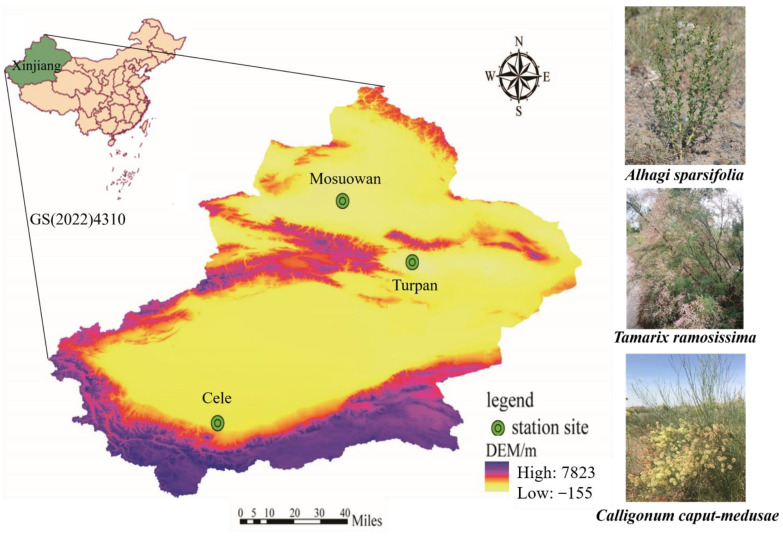
The three sampling sites at Cele, Turpan, and Mosuowan are located in Tarim Basin, Turpan Basin, and Junggar Basin, respectively.

The experiment took place at three desert locations, namely Cele Desert Research Station (Cele, CL; Taklimakan desert), Turpan Desert Botanical Garden (Turpan, TLF; Kumtag desert), and Mosuowan Desert Research Station (Mosuowan, MSW; Gurbantünggüt desert) (Figure 8 and Table 4).

In each of the three research sites, four homogeneous quadrats (each about 30 m × 30 m) were chosen, each with three types of desert plants (*A. sparsifolia*, *T. ramosissima*, and *C. caput-medusae*) (species that grow roughly the same) that exhibit strong development in their native environment [81]. A total of 12 research blocks were selected for this study. In 2022, field research was carried out during the spring (May), summer (July), and autumn (September) at three different long-term monitoring sites to gather root endosphere (RE), rhizosphere soil (RS), and bulk soil (BS) samples from the three desert plants.

### 4.2. Sample Collection from Different Compartments for Microbial Analysis

Soil samples were collected from a depth of 0 to 2 m, and the collection of root samples was evenly distributed within this depth range. We use the detailed approach of Edwards (2015) [82] to differentiate compartments {RE, RS, and BS}. To mitigate the risk of contamination, gloves were replaced and hand sanitization was performed with cotton swabs saturated in alcohol. Fine roots were employed for the examination of both physical and chemical properties through the extraction of loosely bound soil (bulk soil compartment was composed of >1 mm of soil tightly adhering to the rhizosphere that was easily shaken from the rhizosphere compartment), while the rhizosphere soil (rhizosphere compartment was composed of ~1 mm of soil tightly adhering to the root surface that was not easily shaken from the root) that was firmly attached to these roots was meticulously gathered by subjecting it to vortex agitation within a sterile centrifuge tube. The roots were submerged in a 95% alcohol solution and underwent three rounds of oscillation, each lasting 15 s. Afterward, the samples underwent three rinses with sterile water, followed by their transfer into sterile centrifuge tubes, which were subsequently stored at a temperature of −80 °C. There were 324 samples (consisting of three species, three compartments, three basins, three seasons, and four replicates) in total for the root-associated microbial communities of three desert plants. There were three species of plants in each quadrat, 36 samples of each plant in each localization, and each plant was treated individually. The samples were stored at −80 °C until the extraction of genomic DNA and subsequent analysis (see Supplementary Materials for detailed methods of determination; Section S1.1 DNA extraction, PCR, and Illumina sequencing; Table 5).

### 4.3. Assessment of the Physical and Chemical Properties of Soil and Root Samples

Soil pH, electrical conductivity (EC), soil organic carbon (SOC), total nitrogen (TN), total phosphorus (TP), total potassium (TK), available nitrogen (AN), available phosphorus (AP), and available potassium (AK) were measured. In root samples, we measured root organic carbon (ROC), TN, TP, and TK (see Supplementary Materials for detailed methods of determination; Section S1.2 Measurement of soil and root physical and chemical properties).

### 4.4. Statistical Analyses

Data analysis was performed using R version 4.1.0 (R Core Team, 2021) [83]. In this study, the ‘stats’, ‘agricolae’, ‘ggplot2’, ‘microeco’, ‘vegan’, and ‘rdacca. hp’ packages were used [84,85,86,87,88]. The relative abundance of bacterial and fungal taxa (top 10 phyla) was determined (using the ggplot2 package). The impact of species and compartments (RE, RS, and BS) on sequencing and OTU number of bacteria and fungi was analyzed using two-way ANOVA. One-way ANOVA was used to assess the variation in species based on OTU number, alpha diversity (Chao1, Shannon, Pielou_e, and Shannon index), root and soil nutrients, and plant growth characteristics (height and crown width) {stats and agricolae packages, using the aov() function, Shapiro–Wilk normality test, least significant difference (LSD) test, mean ± standard error, and *p* < 0.05} [87]. Beta diversity was represented using NMDS (non-metric multidimensional scaling) and the Bray–Curtis dissimilarity matrix, conducting PERMANOVA (permutational multivariate analysis of variance) (using the vegan package, stress values, ANOSIM, and ADONIS tests) [86]. The core and differential microbiota of bacteria and fungi (at the OTU level) (RE, RS, and BS) in different desert plants were compared and visualized using the microeco and ggplot2 packages, respectively. Utilizing the microeco package, a LEfSe analysis (linear discriminant analysis effect size) was executed to identify specific biomarkers indicative of phylum-level variations in bacterial and fungal communities (RE, RS, and BS) across diverse desert plant species [84]. Gephi software (version 0.9.2) was employed to construct co-occurrence networks, utilizing Spearman’s correlation matrix that featured an absolute correlation coefficient exceeding 0.7 and an FDR-adjusted (false discovery rate) value below 0.001. Furthermore, redundancy analysis (RDA) was conducted with the vegan and rdacca.hp packages to explore the relationship between the microbial communities {bacterial and fungal (RE, RS, and BS), at the OTU level} associated with environmental factors (soil and root nutrients) [85,88].

## 5. Conclusions

In our research, it was found that *A. sparsifolia*’s plant height and crown width were significantly lower than those of *T. ramosissima* and *C. caput-medusae*, but it had the highest soil and root nutrient levels. Furthermore, the RS (rhizosphere soil) and BS (bulk soil) bacterial and fungal diversity of *A. sparsifolia* was significantly lower but increased in RE (rhizosphere endosphere). Conversely, *T. ramosissima* and *C. caput-medusae* showed a high bacterial and fungal variety in RS and BS, while having less diversity in RE. At the same time, the lower the OTU number in the three compartments (RE, RS, and BS), the higher the complexity of the network; conversely, the higher the OTU number, the smaller the complexity of the network. Significant differences were observed in the root-associated (RE, RS, and BS) bacterial and fungal communities due to the combined effects of root nutrients and soil physical and chemical properties. Safeguarding the synergistic development of pivotal microbial populations and host plants is crucial for alleviating the negative impacts of future global environmental changes on desert plants.

## Figures and Tables

**Table 1:** Soil physical and chemical properties.

Index	*A. sparsifolia*	*T. ramosissima*	*C. caput-medusae*
SOC (g·kg^−1^)	3.05 ± 0.24 a	3.59 ± 0.35 a	1.59 ± 0.08 b
TN (g·kg^−1^)	0.25 ± 0.02 a	0.32 ± 0.04 a	0.12 ± 0.01 b
TP (g·kg^−1^)	0.80 ± 0.03 a	0.80 ± 0.03 a	0.65 ± 0.04 b
TK (g·kg^−1^)	19.64 ± 0.19 a	20.39 ± 0.52 a	19.74 ± 0.23 a
AN (mg·kg^−1^)	16.83 ± 1.32 b	22.03 ± 1.5 a	6.97 ± 0.47 c
AP (mg·kg^−1^)	3.42 ± 0.39 b	4.48 ± 0.36 a	2.30 ± 0.10 c
AK (mg·kg^−1^)	344.58 ± 33.11 a	304.14 ± 18.18 a	198.19 ± 8.26 b
pH	8.68 ± 0.05 b	8.58 ± 0.02 b	8.89 ± 0.05 a
EC (μs·cm^−1^)	1831.36 ± 257.43 a	1137.28 ± 160.66 b	343.24 ± 37.04 c

Note: Different lowercase letters (a, b, and c) indicate that the different plants have significant differences (LSD test, *p* < 0.05). EC, electrical conductivity; SOC, soil organic carbon; TN, total nitrogen; TP, total phosphorus; TK, total potassium; AN, available nitrogen; AP, available phosphorus; AK, available potassium.

**Table 2:** Root physical and chemical properties.

Index	*A. sparsifolia*	*T. ramosissima*	*C. caput-medusae*
ROC (g·kg^−1^)	462.37 ± 2.70 b	446.67 ± 2.37 c	471.82 ± 3.42 a
TN (g·kg^−1^)	11.68 ± 0.35 a	4.37 ± 0.17 c	7.80 ± 0.51 b
TP (g·kg^−1^)	0.56 ± 0.06 a	0.22 ± 0.03 b	0.57 ± 0.07 a
TK (g·kg^−1^)	6.89 ± 0.29 a	2.53 ± 0.09 c	3.34 ± 0.12 b

Note: Different lowercase letters (a, b, and c) indicate that the same site and plants and different seasons have significant differences (LSD test, *p* < 0.05). ROC, root organic carbon; TN, total nitrogen; TP, total phosphorus; TK, total potassium.

**Table 3:** Effects of species, compartments, and their interactions on sequencing and OTU number.

	Index	Species	Compartment	Species × Compartment
Bacteria	Sequencing number	6.17 **	26.49 ***	2.29 **
	OTU number	6.60 **	114.86 ***	16.06 ***
Fungi	Sequencing number	9.06 ***	36.25 ***	9.99 ***
	OTU number	16.40 ***	60.49 ***	1.15

Note: Values indicate results of F value. ** *p* < 0.01; *** *p* < 0.001.

**Table 4:** Geographic and climatic characteristics in the three study sites {Xinjiang Institute of Ecology and Geography, Chinese Academy of Sciences (XIEG, CAS)}.

Characteristics		Site		
		Cele	Mosuowan	Turpan
Geographic	Latitude (° N)	35°00′57″	45°07′27″	42°51′59″
	Longitude (° E)	80°43′45″	86°01′31″	89°12′01″
Climatic	MAT (°C)	11.9	6.6	13.9
	MAP (mm)	35.1	117.0	16.4
	PEP (mm)	2595.3	1979.5	3000
	AI	0.01	0.06	0.005
Soil type	ST	aeolian sandy soil	gray desert soil	grayish brown desert soil

Note: MAT, mean annual temperature; MAP, mean annual precipitation; PEP, potential evapotranspiration; AI, aridity index, calculated as AI = MAP/PEP; ST, soil type.

**Table 5:** Primer sets and thermal profiles used in PCR amplification.

Target Group	Primer	Sequence (5′–3′)	PCR Conditions
Bacterial 16S_V3V4	341F	CCTAYGGGRBGCASCAG	All PCR reactions were carried out with 15 µL of Phusion^®^ High-Fidelity PCR Master Mix (New England Biolabs, Ipswich, MA, USA), 0.2 µM of forward and reverse primers, and about 10 ng template DNA. Thermal cycling consisted of initial denaturation at 98 °C for 1 min, followed by 30 cycles of denaturation at 98 °C for 10 s, annealing at 50 °C for 30 s, and elongation at 72 °C for 30 s and 72 °C for 5 min.
	806R	GGACTACNNGGGTATCT AAT	
Fungal ITS_1-5 F	5-1737F	GGAAGTAAAAGTCGTAACAAGG	
	2-2043R	GCTGCGTTCTTCATCGATGC	

## Data Availability

For any data requests, please contact the first and corresponding authors directly.

## Supplementary Materials

The following supporting information can be downloaded at https://www.mdpi.com/article/10.3390/plants13172468/s1. Figure S1: Sequence number of root endosphere, rhizosphere soil, and bulk soil bacteria and fungi in three desert plants; Figure S2: Alpha diversity (Chao1, Shannon, Pielou_e, and Simpson index) of root endosphere, rhizosphere soil, and bulk soil fungi in three desert plants; Figure S3: Beta diversity (Bray–Curtis) of root endosphere, rhizosphere soil, and bulk soil fungi of three desert plants; Figure S4: Co-occurrence network of root endosphere, rhizosphere soil, and bulk soil fungi of three desert plants; Table S1: Plant growth characteristics; Table S2: Bare soil physical and chemical properties. References [89,90,91,92,93,94,95,96,97,98,99,100] are cited in the supplementary materials.
