# Supplementary material for: Variation in Root-Associated Microbial Communities among Three Different Plant Species in Natural Desert Ecosystem
Source: Plants (Basel). 2024 Sep 3;13(17):2468. doi: 10.3390/plants13172468 (PMC11396838; doi:10.3390/plants13172468)
Supplement: Supplementary file 1 [file plants-13-02468-s001.zip › plants-3137264-supplementary.pdf]

Supplementary information

Variation in Root-associated Microbial Communities among Three Different Plant Species in Natural Desert Ecosystem

Yulin Zhang[1,2,3,4], Yi Du[2,3,4,5], Zhihao Zhang [2,3,4], Waqar Islam[2,3,4] *, Fanjiang Zeng[1,2,3,4,5]*

[1] College of Ecology and Environmental, Xinjiang University, Urumqi 830046, China

[2] Xinjiang Key Laboratory of Desert Plant Roots Ecology and Vegetation Restoration, Xinjiang Institute of Ecology and Geography, Chinese Academy of Sciences, Urumqi 830011, China

[3] State Key Laboratory of Desert and Oasis Ecology, Key Laboratory of Ecological Safety and Sustainable Development in Arid Lands, Xinjiang Institute of Ecology and Geography, Chinese Academy of Sciences, Urumqi 830011, China

[4] Cele National Station of Observation and Research for Desert-Grassland Ecosystems, Cele 848300, China

[5] University of Chinese Academy of Sciences, Beijing 100049, China

* Corresponding author.

E-mail addresses:

ddoapsial@yahoo.com / waqarislam@ms.xjb.ac.cn (**Waqar Islam**)

zengfj@ms.xjb.ac.cn (**Fanjiang Zeng**)

S1.1 DNA extraction, PCR, and Illumina sequencing

The DNeasy Power Soil DNA Isolation Kit (Qiagen, Inc., Netherlands) was used to extract total microbial DNA from 0.5 g of BS and RS, following the provided instructions. For root samples, 0.4 g was first ground with liquid nitrogen, and then the DNeasy Plant Maxi Kit (Qiagen, Inc., Netherlands) was applied to extract total microbial DNA. The quantity of the obtained DNA was determined using the NanoDrop ND-1000 spectrophotometer (Thermo Fisher Scientific, Waltham, MA, USA), and its quality was assessed through agarose gel electrophoresis. The PCR (Polymerase Chain Reaction) products were purified using agarose gel electrophoresis with a 2% concentration. Post-detection, the qualified PCR products underwent purification utilizing magnetic beads and were subsequently quantified using a microplate reader. Based on the PCR product concentration, the same amount of samples were mixed. After fully mixing, the PCR products underwent further detection via 2% agarose gel electrophoresis. Subsequently, the bands were retrieved utilizing the glue recovery kit supplied (made in Qiagen, Netherlands). The library was built using the TruSeq® DNA PCR-Free Sample Preparation Kit, followed by quantification using Qubit and Q-PCR. Following library qualification, NovaSeq 6000 was used for on-machine sequencing.

According to the Barcode sequence and PCR amplified primer sequence, each sample data was separated from the dismounting data. After the Barcode sequence and primer sequence were cut off, the reads of each sample were spliced using FLASH (v1.2.11) software [1]. The spliced sequences were Raw Tags data. Then FASTP (v0.23.1) software is used to process the Raw Tags obtained by splicing through strict filtering to obtain high-quality Clean Tags data [2]. The Tags obtained after the above processing need to be processed to remove the chimeric sequence. The Tags sequence is compared with the species annotation database through VSEARCH (v2.16.0) software to detect the chimeric sequence, and the chimeric sequence is finally removed to obtain the final Effective Tags [3].

Additional sequence filtering was performed using the QIIME-II software (v202202) [4]. Operational taxonomic units (OTUs) were generated using the UPARSE software (v7.0.1001) with a 97% similarity threshold [5]. Bacterial OTUs were classified with an RDP classifier (confidence threshold of 70%) based on the Mothur method and SSUrRNA database of Silva database (version 138.1) (Set threshold to 0.8~1) [6-7]. Fungal OTUs were classified based on the UNITE database [8] were utilized. To ensure bacterial and fungal (RE, RS, and BS) sequence uniformity among all samples, the minimum number (174 and 29166) of sequences {dataset$sample_sums () %>% range} were used as the depth to filter other samples to generate in a filtered OTU table {This result removed plant sequences ("mitochondria", "chloroplasts") in fungal results, however,

bacteria do not remove plant sequences}. The raw sequencing data are available at the National Center for Biotechnology Information (NCBI) Short Read Archive, BioProjectID PRJNA1024038.

1. Magoč, T.; Salzberg, S.L. FLASH: fast length adjustment of short reads to improve genome assemblies. *Bioinformatics*, **2011**, 27, 2957-2963. [CrossRef] [PubMed]
2. Bokulich, N.A.; Subramanian, S.; Faith, J.J.; Gevers, D.; Gordon, J.I.; Knight, R.; Mills, D.A.; Caporaso, J.G. Quality-filtering vastly improves diversity estimates from Illumina amplicon sequencing. *Nat Methods*, **2013**, 10, 57-59. [CrossRef] [PubMed]
3. Rognes, T.; Flouri, T.; Nichols, B.; Quince, C.; Mahé, F. VSEARCH: a versatile open source tool for metagenomics. *PeerJ*, **2016**, 4, e2584. [CrossRef] [PubMed]
4. Caporaso, J.G.; Kuczynski, J.; Stombaugh, J.; Bittinger, K.; Bushman, F.D.; Costello, E.K.; Fierer, N.; Peña, A.G.; Goodrich, J.K.; Gordon, J.I.; Huttley, G.A.; Kelley, S.T.; Knights, D.; Koenig, J.E.; Ley, R.E.; Lozupone, C.A.; McDonald, D.; Muegge, B.D.; Pirrung, M.; Reeder, J.; Sevinsky, J.R.; Turnbaugh, P.J.; Walters, W.A.; Widmann, J.; Yatsunenko, T.; Zaneveld, J.; Knight, R. QIIME allows analysis of high-throughput community sequencing data. *Nat Methods*, **2010**, 7, 335-6. [CrossRef] [PubMed]
5. Edgar, R.C. UPARSE: highly accurate OTU sequences from microbial amplicon reads. *Nat methods*, **2013**, 10, 996-998. [CrossRef] [PubMed]
6. Wang, Q.; Garrity, G.M.; Tiedje, J.M.; Cole, J.R. Naive Bayesian classifier for rapid assignment of rRNA sequences into the new bacterial taxonomy. *Appl Environ Microbiol*, **2007**, 73, 5261-5267. [CrossRef] [PubMed]
7. Quast, C.; Pruesse, E.; Yilmaz, P.; Gerken, J.; Schweer, T.; Yarza, P.; Peplies, J.; Glöckner, F.O. The SILVA ribosomal RNA gene database project: improved data processing and web-based tools. *Nucleic Acids Res*, **2013**, 41, D590-D596. [CrossRef] [PubMed]
8. Abarenkov, Kessy.; Nilsson, R.H.; Karl-Henrik, L.; Taylor, A.F.S.; May, T.W.; Frøslev, T.G.; Pawlowska, J.; Lindahl, B.; Põldmaa, K.; Truong, C.; Vu, D.; Hosoya, T.; Niskanen, T.; Piirmann, T.; Ivanov, F.; Zirk, A.; Peterson, M.; Cheeke, T.E.; Ishigami, Y.; Jansson, A.T.; Jeppesen, T.S.; Kristiansson, E.; Mikryukov, V.; Miller, J.T.; Oono, R.; Ossandon, F.J.; Paupério, J.; Saar, I.; Schigel, D.; Suija, A.; Tedersoo, L.; Kõljalg, U. The UNITE database for molecular identification and taxonomic communication of fungi and other eukaryotes: sequences, taxa and classifications reconsidered. *Nucleic Acids Res*, **2024**, 52, D791-D797. [CrossRef] [PubMed]

S1.2 Measurement of soil and root physicochemical properties

To measure soil and root organic carbon (SOC and ROC), total nitrogen (TN), available nitrogen (AN), and available potassium (AK), the following methods were used: the $K_2Cr_2O_7$-H_2SO_4 oxidation method, the Kjeldahl Nitrogen Analyzer (K1160, Jinan Hanon Instruments Co. Ltd., China), the alkali hydrolyzable method, and the NH_4OAc extraction method [9-11]. Before measuring total phosphorus (TP) and total potassium (TK) with the Inductively Coupled Plasma-Optical Emission Spectrometer (iCAP 6300, Thermo Elemental, USA), the samples were digested in concentrated HNO_3 [12]. To determine the amount of accessible phosphorus (AP), a continuous-flow autoanalyzer (Autoanalyzer 3, Bran and Luebbe, Germany) with HCl/NH_4F was used to perform colorimetric analysis using the ascorbic acid molybdate technique [10, 11]. The pH of the soil {soil/water ratio of 1:2.5 (w/v)} and its electrical conductivity (EC) {soil/water ratio of 1:5 (w/v)} were measured using a pH and EC meter (PHSJ-6 L and DDSJ-319 L, INESA Scientific Instrument Co. Ltd. in China) manufactured, respectively.

9 Hooper, D.U.; Vitousek, P.M. Effects of plant composition and diversity on nutrient cycling. *Ecol Monogr*, **1998**, 68, 121–149. [CrossRef]

10 Neff, J.C.; Reynolds, R.L.; Belnap, J.; Lamothe, P.J. Multi-decadal impacts of grazing on soil physical and biogeochemical properties in southeast Utah. *Ecol Appl*, **2005**, 15, 87–95. [CrossRef]

11 Warra, H.H.; Ahmed, M.A.; Nicolau, M.D. Impact of land cover changes and topography on soil quality in the Kasso catchment, Bale Mountains of southeastern Ethiopia. *Singapore Journal of Tropical Geography*, **2015**, 36, 357–375. [CrossRef]

12 Lu, X.Y.; Yan, Y.; Sun, J.; Zhang, X.K.; Chen, Y.C.; Wang, X.D.; Cheng, G.W. Carbon, nitrogen, and phosphorus storage in alpine grassland ecosystems of Tibet: Effects of grazing exclusion. *Ecol Evol*, **2015**, 5, 4492–4504. [CrossRef] [PubMed]

Table S1 Plant growth characteristics

**Species**	Height (cm)	Crown width (cm^2)
A. sparsifolia	46.25±3.22c	2705.17±531.59c
T. ramosissima	202.08±10.74b	41745.08±5967.57b
C. caput-medusae	248.92±14.53a	74213.67±7566.51a

Note: Different lowercase letters (a, b, and c) indicate that the different desert plants have significant differences (LSD test, $P < 0.05$).

Table S2 Bare soil physical and chemical properties

Site	pH	EC (μs•cm^{-1})	TN (g•kg^{-1})	TP (g•kg^{-1})	TK (g•kg^{-1})
CL	8.46±0.07b	323.50±22.99b	0.15±0.01a	0.57±0.01a	19.54±0.10a
TLF	8.63±0.01a	480.50±12.24a	0.10±0.001c	0.54±0.01b	18.51±0.13b
MSW	8.51±0.05ab	505.00±34.66a	0.13±0.003b	0.54±0.01b	18.88±0.21b

Site	SOC (g•kg^{-1})	AN (mg•kg^{-1})	AP (mg•kg^{-1})	AK (mg•kg^{-1})
CL	1.72±0.09a	4.40±0.30a	0.54±0.03b	57.50±1.04c
TLF	1.24±0.04b	4.20±0.13a	1.77±0.30a	118.00±2.48b
MSW	1.42±0.02b	4.95±0.30a	1.98±0.29a	144.50±2.02a

Note: Different lowercase letters (a, b, and c) indicate that the different sites have significant differences (LSD test, $P < 0.05$). **EC**, electrical conductivity; **SOC**, soil organic carbon; **TN**, total nitrogen; **TP**, total phosphorus; **TK**, total potassium; **AN**, available nitrogen; **AP**, available phosphorus; **AK**, available potassium.

Figure legends

Figure. S1 Sequence number [A (number of sequence) of the bacteria] and [B (number of sequence) of the fungi)] of root endosphere (RE), rhizosphere soil (RS), and bulk soil (BS) bacteria and fungi in three desert plants.

Figure. S2 Alpha diversity {[A, B, and C (Chao1), [D, E, and F (Shannon)], [G, H, and I (Pielou_e)], and [J, K, and L (Simpson)] indexes} of root endosphere (RE), rhizosphere soil (RS), and bulk soil (BS) fungi in three desert plants. Different lowercase letters (a and b) indicate significant differences among species at the $p < 0.05$ level and the ns indicate no significant differences among species at the $p > 0.05$ level (ANOVA and Duncan's test).

Figure. S3 Beta diversity {[A, B, and C (Bray–Curtis)] and [D, E, and F (nonmetric multidimensional scaling)]} of root endosphere (RE), rhizosphere soil (RS), and bulk soil (BS) fungi of three desert plants. ** $p < 0.01$.

Figure. S4 Co-occurrence network {[A, D, and G (Network characteristics)] of the *A. sparsifolia*, [B, E, and H (Network characteristics)] of the *T. ramosissima*, and [C, F, and I (Network characteristics)] of the *C. caput-medusae*} of root endosphere (RE), rhizosphere soil (RS), and bulk soil (BS) fungi of three desert plants.

Figure S1

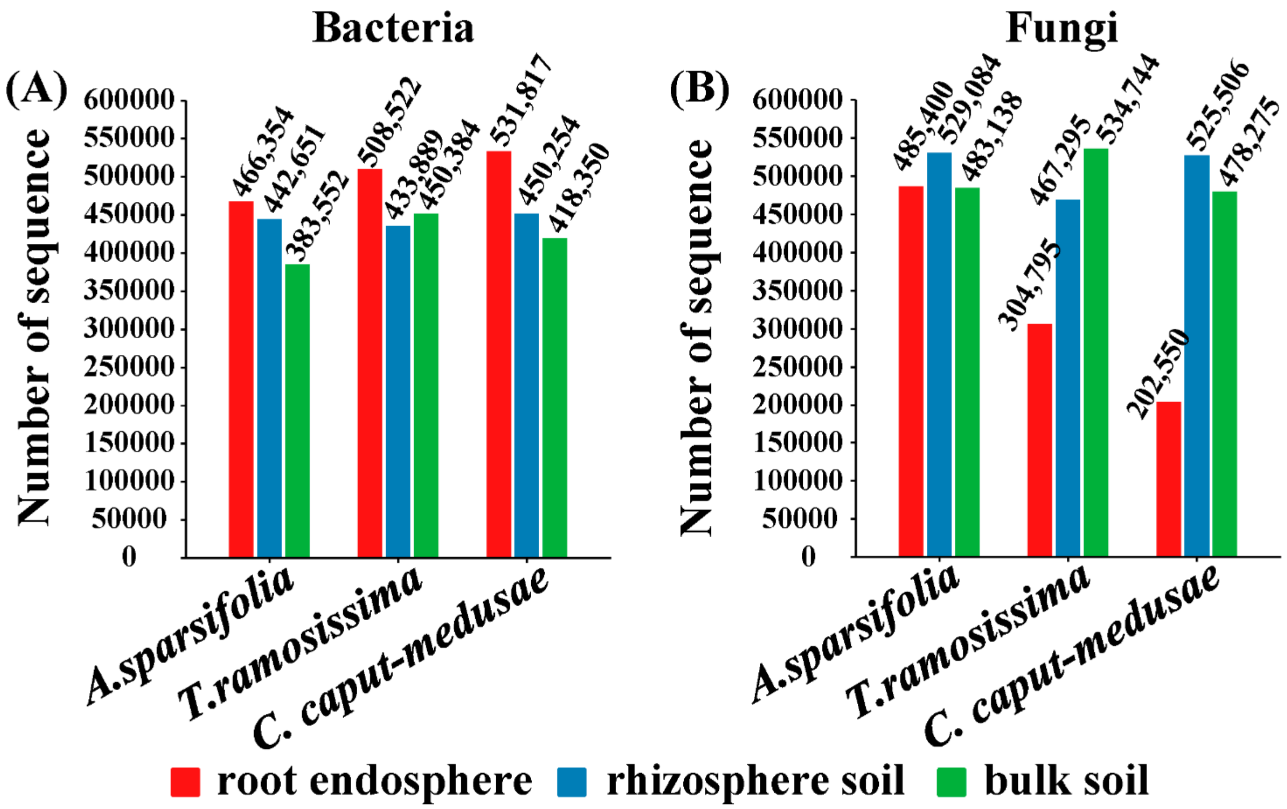

Bacteria
Fungi
(A)
(B)
Number of sequence
600000
550000
500000
450000
400000
350000
300000
250000
200000
150000
100000
50000
0
466,354
442,651
383,552
508,522
433,889
450,384
531,817
450,254
418,350
485,400
529,084
483,138
304,795
467,295
534,744
202,550
525,506
478,275
A.sparsifolia
T.ramosissima
C. caput-medusae
root endosphere
rhizosphere soil
bulk soil

Figure S2

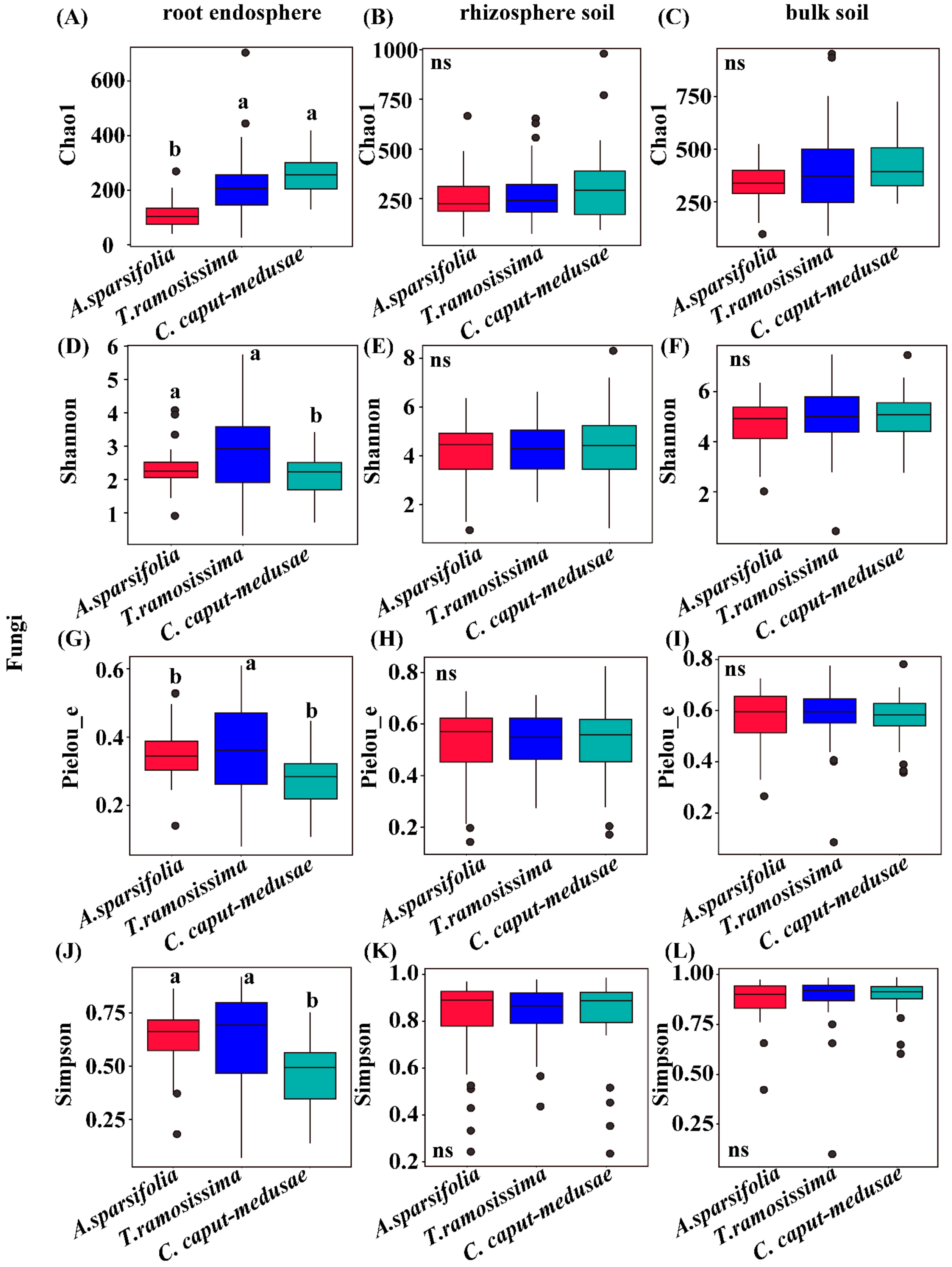

(A)
root endosphere
(B)
rhizosphere soil
(C)
bulk soil
Fungi
Chao1
Shannon
Pielou_e
Simpson
(D)
(E)
(F)
(G)
(H)
(I)
(J)
(K)
(L)
ns
A.sparsifolia
T.ramosissima
C. caput-medusae

Figure S3

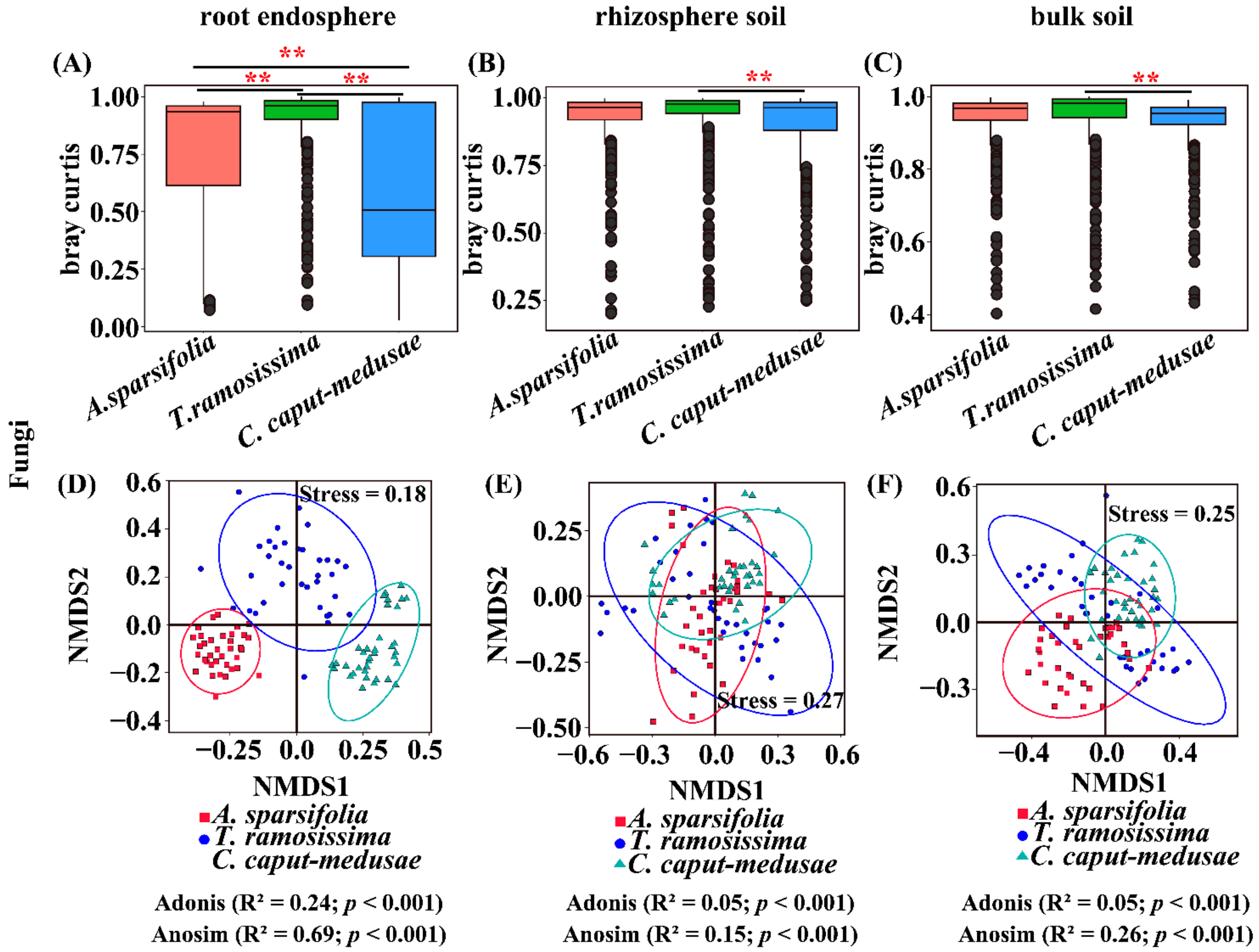

root endosphere
rhizosphere soil
bulk soil
Fungi
(A)
(B)
(C)
**
bray curtis
1.00
0.75
0.50
0.25
0.00
0.8
0.6
0.4
1.0
A.sparsifolia
T.ramosissima
C. caput-medusae
(D)
(E)
(F)
Stress = 0.18
Stress = 0.27
Stress = 0.25
NMDS2
NMDS1
A. sparsifolia
T. ramosissima
C. caput-medusae
Adonis (R^2 = 0.24; p < 0.001)
Anosim (R^2 = 0.69; p < 0.001)
Adonis (R^2 = 0.05; p < 0.001)
Anosim (R^2 = 0.15; p < 0.001)
Adonis (R^2 = 0.05; p < 0.001)
Anosim (R^2 = 0.26; p < 0.001)

Figure S4

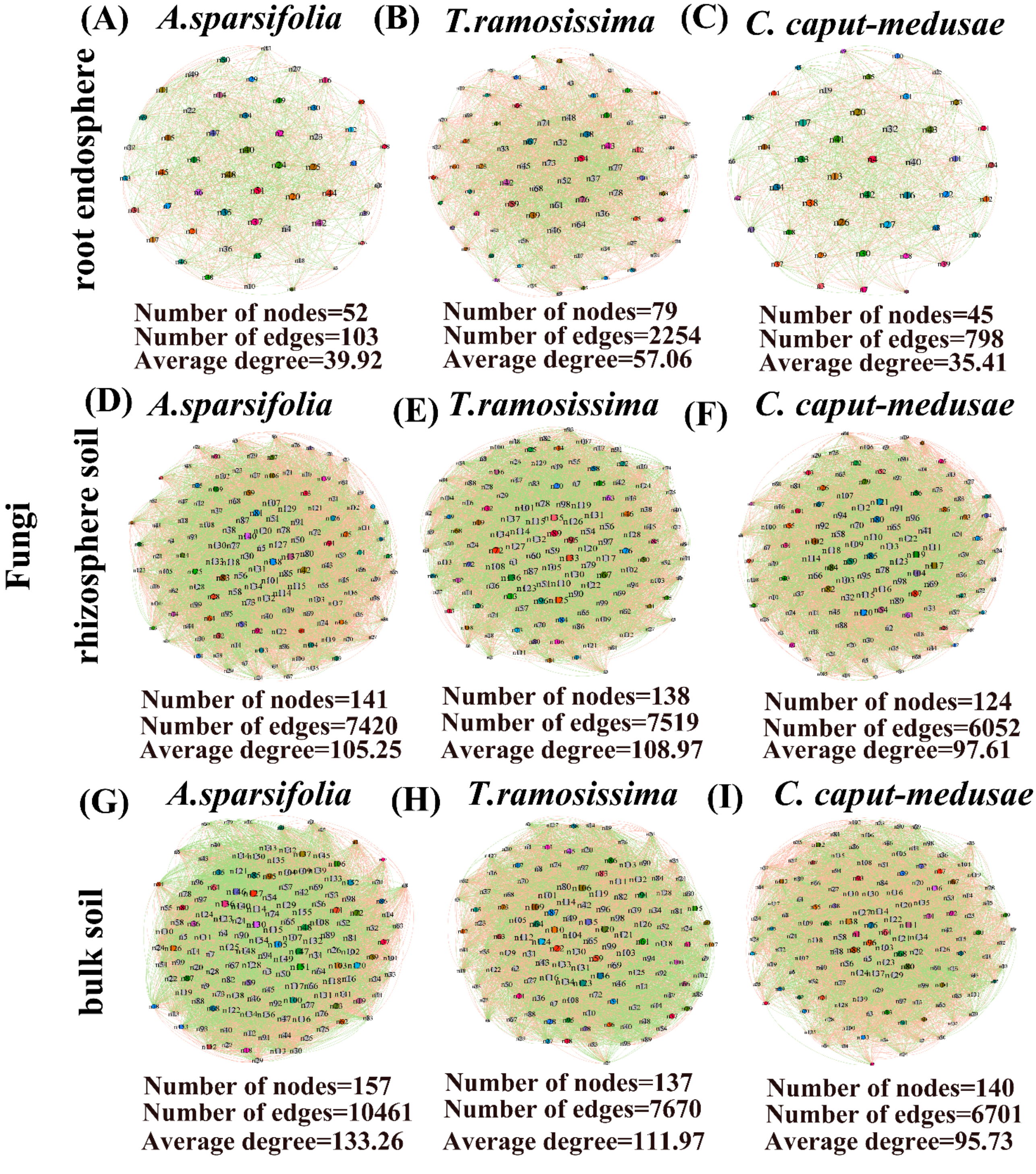
Fungi
root endosphere
rhizosphere soil
bulk soil
(A) A.sparsifolia
(B) T.ramosissima
(C) C. caput-medusae
Number of nodes=52
Number of edges=103
Average degree=39.92
Number of nodes=79
Number of edges=2254
Average degree=57.06
Number of nodes=45
Number of edges=798
Average degree=35.41
(D) A.sparsifolia
(E) T.ramosissima
(F) C. caput-medusae
Number of nodes=141
Number of edges=7420
Average degree=105.25
Number of nodes=138
Number of edges=7519
Average degree=108.97
Number of nodes=124
Number of edges=6052
Average degree=97.61
(G) A.sparsifolia
(H) T.ramosissima
(I) C. caput-medusae
Number of nodes=157
Number of edges=10461
Average degree=133.26
Number of nodes=137
Number of edges=7670
Average degree=111.97
Number of nodes=140
Number of edges=6701
Average degree=95.73
